# Living on a Thread: A Case of Critical Left Main Coronary Artery Disease With an Unusual Presentation

**DOI:** 10.7759/cureus.26942

**Published:** 2022-07-17

**Authors:** Issa Asfour, Manar Jbara

**Affiliations:** 1 Internal Medicine, East Tennessee State University, Johnson City, USA; 2 Cardiology, East Tennessee State University, Johnson City, USA

**Keywords:** atypical symptoms, cardiac troponin, coronary artery bypass grafting(cabg), cardio vascular disease, left main coronary artery disease, significant coronary artery disease

## Abstract

The left main disease is significant stenosis of the left coronary artery, which is responsible of supplying blood to a major portion of the left ventricle. In this report, we describe a unique case of critical left main disease with a special clinical presentation.

A 66-year-old male with insignificant past medical history presented with dyspepsia. Patient presented twice to the emergency department seeking for help for his persistent complaint. During his second visit, patient was diagnosed with type one myocardial infarction and underwent coronary angiography which showed 90% stenosis in the left main coronary artery. Patient underwent successful coronary artery bypass grafting and was sent home.

This case is a unique representation of type 1 myocardial infarction as the peak troponin I level does not correlate with the extent of the myocardium being jeopardized. A big portion of the heart is at risk of injury with the 90% stenosis found in this patient’s left main coronary artery, yet the peak troponin I level is minimum. This report provides a possible explanation of the discrepancy between the peak troponin I level and the extent of the myocardium being jeopardized and describes a common yet easily missed clinical presentation of acute coronary syndrome.

Left main disease is a relatively uncommon presentation of acute coronary syndrome, with potentially serious detrimental consequences. Discrepancies do occur among patients of critical left main disease, and promptly diagnosing and managing is of great importance.

## Introduction

Acute coronary syndrome is one of the most common heart pathologies worldwide [1]. It is due to an inadequate supply of blood and oxygen to the myocardium, secondary to occlusion of the coronary arteries, which results in a demand-supply mismatch and eventual myocardial damage [2]. It is considered a major cause of mortality and morbidity worldwide, and promptly diagnosing and treating it are particularly important [2].

Severe left main disease occurs in around three to seven percent of all patients undergoing diagnostic coronary angiography [3,4]. The left main disease is a significant narrowing of the left main coronary artery, which supplies more than two-thirds of the left ventricle in patients with a right dominant circulatory system. It is associated with a poor prognosis and has a detrimental effect on the heart [5]. Cardiac troponin measurements are considered the preferred diagnostic markers of acute myocardial injury [6].

In this report, we describe a case of critical left main disease with a peak troponin I level that does not reflect the extent of the myocardium that is being jeopardized.

## Case presentation

**Figure 1:**
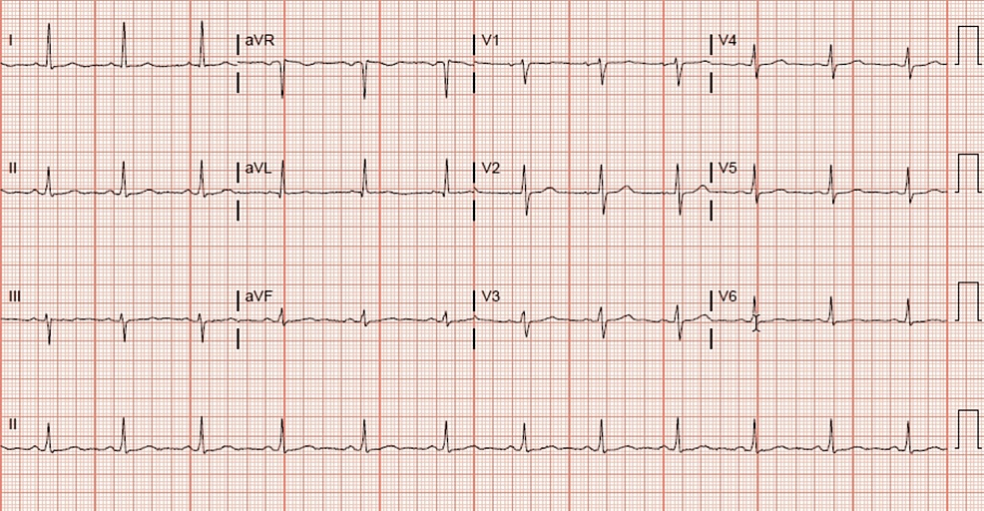
Non-specific ST changes with no acute ischemic changes

**Figure 2:**
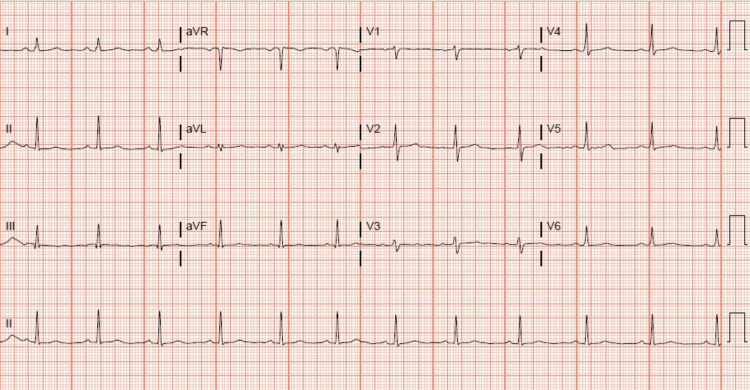
non-specific ST changes with no acute ischemic changes

**Figure 3:**
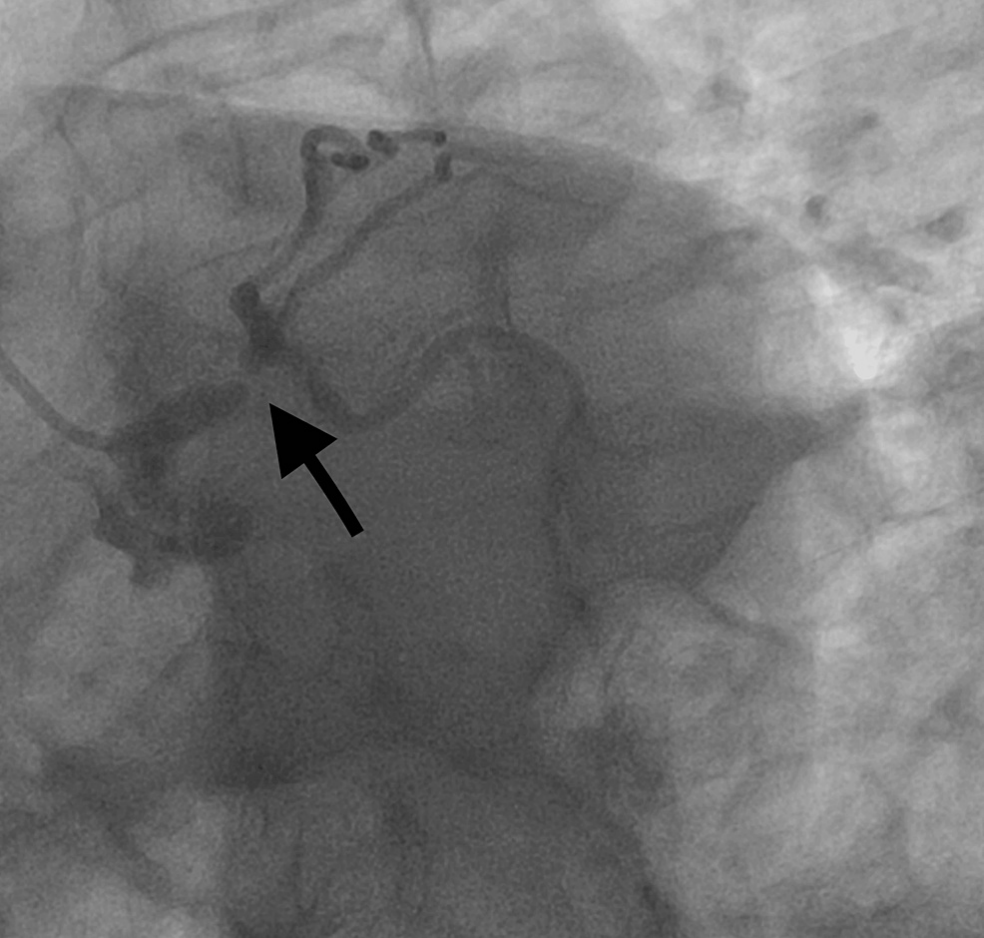
Distal left main coronary artery stenosis of 90%

A 66-year-old male with an insignificant past medical history presented to the emergency department with dyspepsia. The patient started having symptoms of dyspepsia early in the morning, which he thought was related to heartburn. He went to the emergency department and his initial work-up, including electrocardiogram (EKG) (Figure 1). His troponin I was not remarkable. The patient then went home, but his dyspepsia symptoms increased in intensity and were associated with exertion. The exertional characteristic of the symptom prompted him to go again to the emergency department seeking help. His physical examination and vital signs were normal. A repeated EKG showed non-specific ST changes with no acute ischemic changes (Figure 2). The results of the laboratory investigation, including a complete blood count, comprehensive metabolic panel, lipid panel, and glycosylated hemoglobin (HbA1c), were all within normal range. Serial cardiac troponin I was drawn three hours apart and the results were as follows: <0.02, 0.06, 0.05, then 0.05 (normal range <0.02 ng/mL). The persistence and exertional characteristics of the patient’s symptoms prompted the start of a heparin drip, aspirin once daily 81 mg, metoprolol tartrate 25 mg twice daily, rosuvastatin 20 mg once daily, and to proceed with inpatient coronary angiography the following day. Cardiac catheterization revealed right dominant circulation with distal left main coronary artery stenosis of 90% (Figure 3). Echocardiogram showed left ventricular ejection fraction of 50-55% and grade 1 diastolic dysfunction with no regional wall motion abnormalities. The patient remained hospitalized and underwent a successful coronary artery bypass grafting without complications. He was safely discharged home on aspirin 81 mg once daily, ticagrelor 90 mg twice daily, rosuvastatin 20 mg once daily, and metoprolol tartrate 25 mg twice daily as per the protocol.

## Discussion

Critical left main coronary artery disease is a clinically important pathology as it is associated with 50% mortality at three-year follow-up [6]. This case is unique as it describes a peak troponin level that is not typical of the degree of the myocardium at risk of injury.

This case represents type one myocardial infarction. According to the Fourth Universal Definition of Myocardial Infarction, type one myocardial infarction is defined by the rise and or drop of troponin I levels with at least one value being above the 99th percentile and a minimum of one of the following: symptoms associated with myocardial ischemia, new EKG changes related to ischemia, new pathological Q waves, imaging suggestive of ischemic etiology including regional wall motion abnormality, and lastly, proof of coronary thrombus through autopsy, angiography or intracoronary imaging [7]. In this case, the patient met the criteria through the rise of troponin I level after a previous normal value in the setting of ongoing symptoms suggestive of acute myocardial ischemia. Myocardial infarction type one is due to atherosclerotic plaque disruption resulting in myocardial damage [7]. 

Our case is special because the patient presented with type 1 myocardial infarction of the left coronary artery, with 90% stenosis in the left main coronary artery, which supplies a major portion of the left ventricle. Yet the peak troponin I level was minimum and did not correlate with the extent of myocardium being at risk of injury. Numerous studies concluded that there is a positive and consistent correlation between peak troponin I level and the infarct size and that it can be used clinically to estimate the infarct size with great precision [8,9].

Two possible explanations that can contribute to the limitation of the peak troponin I level to accurately estimate the infarction size in this case. First, troponin I level concentration varies throughout the myocardium tissue; it is lowest in the atrium and highest in the ventricles [10]. Second, the troponin I levels in the ventricle are uniform, however, there are some person-to-person discrepancies [10]. These findings suggest that measuring peak troponin I levels would never provide optimal accuracy with regard to estimating the infarct size.

Another finding in this case that has an educational clinical significance is the patient’s presentation. The patient had type 1 myocardial infarction with 90% stenosis of the left main coronary artery, which put a big portion of the heart at risk of injury. Yet he presented with minimum clinical symptoms. This presentation, even though not uncommon in coronary artery syndrome, can be easily missed, as was in the case of this patient, who had to seek help twice before receiving the proper management. The delay in reaching a diagnosis could have resulted in fatal consequences.

## Conclusions

Left main disease is considered a rare presentation of acute coronary syndrome. This case represents a unique description of the presentation of this important pathology. It calls for the need to further study this pathology to promptly diagnose and manage it and avoid fatal consequences secondary to delay or misdiagnosis, especially, as shown in this case. 
